# A qualitative study of persons who inject drugs but who have never helped others with first injections: how their views on helping contrast with the views of persons who have helped with first injections, and implications for interventions

**DOI:** 10.1186/s12954-018-0250-x

**Published:** 2018-08-28

**Authors:** David M. Barnes, Don C. Des Jarlais, Margaret Wolff, Jonathan Feelemyer, Susan Tross

**Affiliations:** 1 Department of Epidemiology, College of Global Public Health, New York University, 665 Broadway, 8th floor, New York, NY 10012 USA; 2 Department of Psychiatry, Icahn School of Medicine at Mount Sinai, 39 Broadway, Suite 530, New York, NY 10006 USA; 3 HIV Center for Clinical and Behavioral Studies, The New York State Psychiatric Institute and Columbia University, 1051 Riverside Drive, Unit 15, New York, NY 10032 USA

**Keywords:** Injection drug use, Drug use interventions, Opioid epidemic, United States of America

## Abstract

**Background:**

Transitioning from non-injection to injection drug use dramatically escalates health risks. Evidence suggests that people who inject drugs (PWID) help in a majority of others’ first injections, yet these helpers represent only a minority of experienced PWID. Recent research has provided insight into this helping process, as reported by helpers. PWID who have never helped, although the majority of PWID, have not previously been the focus of study. To address this gap, we give primary voice to non-helpers’ perspectives on the helping process, while also comparing their views with persons in our sample who have helped with first injections. Finally, we consider how non-helpers’ perspectives can inform harm reduction interventions to reduce, or make safer, initiation into injecting drug use.

**Methods:**

We conducted audio-recorded, qualitative interviews with 23 current opioid injectors on Staten Island, NY, where the opioid epidemic is pronounced. Seventeen had never helped with first injections and 6 had. Interviews were transcribed verbatim, and three coders used a consensus-developed codebook to code all interviews. Framework analysis was used to identify overarching themes.

**Results:**

We identified three key themes in non-helpers’ discourse around not helping: altruistic motivations to prevent immediate and delayed harms to individuals injecting for the first time; inhibition due to negative assessments of their own injecting skills; and absolutist ethical convictions against helping. Non-helpers differed from helpers on each theme.

**Conclusions:**

Because most PWID have never helped with first injections, their perspectives on helping warrant consideration and can inform harm reduction interventions to reduce, or make safer, transitions to injection drug use. Their perspectives can be used to broaden the factors PWID consider around questions of promoting injection and helping with others’ first injections, including considerations of the moral issues involved in choosing to help or not to help.

## Background

Transitioning from non-injection to injection drug use can precipitate a cascade of negative health [1–5], social, and economic [3, 6, 7] consequences for the new injector. HIV and Hepatitis C infection (HCV), skin abscesses, and overdose are among the most serious health consequences.

In USA’s current opioid epidemic, a shift has occurred over the last decade from extra-medical opioid analgesic use to heroin [6, 8–13], and from sniffing to injecting heroin [2, 6, 14–16]. A still more recent trend involves adulteration of heroin with fentanyl [17], an opioid significantly more potent than heroin. These shifts have generated dramatic increases in overdose, overdose death, and HCV infection in the USA since 2010 [5, 9, 18–22].

In light of evidence suggesting burgeoning [23, 24] and increasingly dangerous injection drug use in the USA, efforts to reduce transitions to injecting are critical. Because drug injection is a learned skill, would-be initiates often require help with first injections from established PWID. In study samples, between 68% [25] and 95% [26] of PWID said experienced PWID helped them with their first injection. However, the percent of PWID endorsing having ever helped with *others*’ first injections is, with one exception [26], consistently below 50% [25, 27–33]. These data indicate that a minority of PWID assist in a majority of transitions to injection.

Recent qualitative [33–35] and quantitative [25, 27–33] studies have examined the circumstances and predictors of experienced PWID initiating others into injection. A frequent finding in the qualitative research is the ambivalence that PWID who have initiated others into injection feel about helping, expressed implicitly by highlighting extenuating circumstances, or explicitly by expressing regret that they had helped with first injections. Examples of extenuating circumstances PWID have described in their accounts of initiating others include first trying to dissuade novices from injecting; characterizing non-injecting users as having already crossed a threshold and therefore sufficiently aware of the risks; describing would-be PWID as autonomous individuals entitled to their own choices; and initiating older but not younger drug users on the grounds that the former have less to lose or are experienced enough to know the risks [33]. PWID who have helped have also explicitly stated regret about having initiated others [34, 35]. The perspectives and behaviors around *not* helping among the majority of PWID who have never assisted has, to date, been given limited attention in the literature [33, 35–38]. Understanding what accounts for not helping among the majority of PWID can be informative to harm reduction interventions that discourage PWID from intentionally or unintentionally fostering injection among non-PWID. If only a minority of PWID have initiated others into injection, and if initiating others triggers ambivalence, then *not* initiating others would seem to be a potentially viable path for an even larger majority of PWID. The present study, using a qualitative approach, is the first that we are aware of to focus on how PWID who have not initiated others into injection think and feel about helping. We compare these views with those of PWID who *have* initiated others, to discern potentially distinct perspectives. Finally, we consider how the non-helping perspective could enrich harm reduction interventions designed to reduce the likelihood of experienced PWID promoting injection and initiating others into injection. We note here, and address at greater length in the “Discussion” section, that the question of helping with others’ first injections raises complex ethical issues.

We distinguish between helping (which we also call initiating) and promoting behaviors. *Helping* behaviors include guiding or explaining while the novice self-injects or actually injecting the novice. The goal of helping behaviors is the novice’s first injection. *Promoting* behaviors include extolling injection (including the more intense “high” achieved from injecting versus other routes of administration), injecting in front of non-injectors, and describing the injection process. Knowingly or not, promoting behaviors can fuel curiosity in injection [28, 36, 38–42]. Interventions to discourage PWID from fostering injection among non-PWID may focus on either or both sets of behaviors. In the interest of depth over breadth, we focus on how the PWID in our sample discuss helping.

We conducted interviews with PWID on Staten Island, one of New York City’s five boroughs, to gain insight into a recent “hotspot” of the opioid epidemic. Between 2011 and 2014, Staten Island had unintentional opioid analgesic overdose death rates 1.8 to 3.2 times higher than the next closest borough, the Bronx, [43], and alternated with the Bronx for the borough with the highest unintentional overdose death rates involving heroin [44]. Of the five boroughs, Staten Island has the smallest population [45], the lowest population density [46], and the second highest median household income (after Manhattan) [47]. It is the only borough where non-Hispanic Whites comprise a majority of the population [48]. In choosing Staten Island, we reasoned that mechanisms sustaining the epidemic would be more apparent in areas where it was particularly active, and therefore our findings could have greater relevance to the opioid epidemic beyond Staten Island.

## Methods

We conducted semi-structured interviews with 23 current PWID, 17 who report having never helped (“non-helpers”), and 6 who report having helped initiate others into injection (“helpers”). Interviews were conducted one-on-one in private spaces in two agencies on Staten Island: a large multi-service community-based organization that is our research partner and a residential substance use treatment program. Eligibility criteria were non-medical opioid injection in the last 2 months; age 18 or older; able to provide informed verbal consent; and English-speaking. At the time of the interviews, study participants were clients of a syringe exchange program (*n* = 22) or in residential treatment (*n* = 1). We instructed program staff to neutrally inform potentially eligible clients of our presence and availability to describe the study to them. This yielded a convenience sample, all but one of whom was recruited from the same target population (i.e., clients of our community partner’s syringe exchange program), and using the same recruitment protocol, of our subsequent study for which these interviews were preparation. Accordingly, we believe our interview sample was representative of our target population, if not representative of PWID in general, and therefore sampling bias was not a concern. DB and ST conducted all semi-structured qualitative interviews and an accompanying survey covering demographics and drug-use histories. We chose to use professional qualitative interviewers, rather than trained peer interviewers, to maximize consistency in following the interview guide, and, because initiating others into injecting drug use is viewed negatively by most PWID, we believed our interviewers were more likely to remain neutral and thereby minimize socially desirable responses in interview participants. Interviews were digitally audio-recorded with the participant’s consent (all 23 consented). Participants were paid $30 in cash for their time and effort. The institutional review board at Mount Sinai Hospital approved all study procedures.

The interview guide elicited accounts, in participants’ own words, of their experiences and reflections around substance use, injection, and initiating others into injection. Specific attention was given to their own drug use history, including their initiation into injection; their experiences of opportunities to help with others’ first injections, including the perceived pros and cons of helping, their likelihood of helping in the future, and the reasons and situations that might make them more likely to help. We classified participants as “helpers” if they said they had ever helped someone with their first injection, ever injected a novice, or if they said they had explained, guided, or demonstrated injecting while the novice self-injected in front of the participant. We classified participants as “non-helpers” if they said they had not engaged in any of these specific helping behaviors.

To develop our interview guide, we drew on the research team’s extensive experience interviewing persons who use substances over several decades, our community collaborators, empirical findings, and on conceptual models and theory. Foremost among empirical findings was the focal role of ambivalence in PWIDs’ attitudes and behaviors related to initiating others into injection [34, 35], which we described above. These empirical findings led us to considerations of the decisional balance model [49, 50] and motivational interviewing [51, 52], since both provide approaches to addressing ambivalence. Ultimately the decisional balance model was more informative because our goal in the interviews was to explore with participants their ambivalence about initiating others into injection, not to promote its resolution or to promote the behavior change of study participants [52]. Accordingly, we crafted open-ended questions to prompt PWID to reflect on the advantages and disadvantages of initiating others into injection that would accrue to themselves and to their initiates. We asked them to reflect on their actual experiences of initiating others, or of choosing not to, the importance they attribute to these choices, and the certainty or ambivalence they feel about their choices. We also drew from the theory of reasoned action [53] to explore PWIDs’ attitudes, perceptions of norms, and their intentions around assisting with others’ first injections *in the future*. To that end, we asked participants how likely they were to initiate others into injecting in the future, and what would make them more, or less, likely to do so. Thus, we hoped to tap into participants’ future intentions to assist, and the personal attitudes and norm perceptions that are proposed in the theory of reasoned action to influence those intentions. Information on these drivers of intention would clearly give us richer contextual information on helping.

Interviews were transcribed verbatim and three trained coders (DB, MW, and JF) coded all interviews using Atlas.ti 8 software (2016, Berlin, Scientific Software Development GmbH). All coders were systematically trained in qualitative coding by ST. The original codebook covered each topic and sub-topic of the interview guide, and was expanded as additional themes emerged during the coding process. All coders separately coded the same four randomly selected interviews and then resolved discrepant coding in subsequent meetings. Records of decision trails for each resolved discrepancy were maintained. Reliability was judged sufficient after these four interviews, and the remaining 19 interviews were coded by one of the three coders. To prevent coding drift, two of these were coded by all three coders following the same protocol as for initial training. Using a framework analytic approach [54], and drawing on existing literature, we charted codes for every participant; identified emergent overarching themes and sub-themes; mapped the co-occurrence of themes and sub-themes, and their clustering in particular participant sub-groups (e.g., individuals who have helped with first injections versus individuals who have not); and interpreted these analyses.

Empirical research findings prompted us during our analysis of the interviews to especially consider four influences on injection helping behavior. First, sexual partnership has been associated with injection initiation, particularly with male partners initiating female partners [30, 55]. Second, a sense of altruism towards non-PWID has been noted [51]. Third, perceptions of one’s own injection skills (positive and negative) [31, 32], have been described both as facilitators of and barriers to injection helping practices. And fourth, others [37] have described social norms in PWID communities against initiating others into injection.

## Results

### Sample characteristics

Table 1 displays demographic and drug-use characteristics of our sample. A majority were male, heterosexual, White, and had at least a high school diploma or GED. All had injected heroin in the last 6 months and a majority injected heroin at least four times per week. Small minorities also injected other drugs. Time injecting ranged from 2 months to 48 years, and half of the sample had injected six or fewer years. Non-helpers were more likely than helpers to be female, 30 or older, and to lack a high school diploma or GED. Also, they injected heroin less frequently and used fewer drugs through non-injecting routes than did helpers. Non-helpers comprised 74% of our sample, which is consistent with other sample proportions [25, 27–33]. The six helpers in our sample had helped a total of 24 individuals with first injections, ranging from 1 to 10 (data not shown). There was no single “type” of person who was helped with a first injection: five helpers said they had helped friends, acquaintances, or strangers, and the sixth helper said he had helped a friend and a “very close companion.”

**Table 1:** Demographic and drug-use characteristics of sample

	Non-helpers (*n* = 17)	Helpers (*n* = 6)	All (*n* = 23)
	*n* (%)	*n* (%)	*n* (%)
Demographics			
Male	8 (47)	5 (83)	13 (57)
Age ≥ 30	9 (53)	2 (33)	11 (48)
Black	3 (18)	0	3 (13)
White	9 (53)	3 (50)	12 (52)
Black-Hispanic	2 (12)	0	2 (9)
White-Hispanic	3 (18)	3 (50)	6 (26)
≥ HS diploma or GED	8 (47)	4 (67)	12 (52)
Heterosexual	14 (82)	5 (83)	19 (83)
Bisexual	2 (12)	1 (17)	3 (13)
Gay or lesbian	1 (6)	0	1 (4)
Drug use last 6 months			
Injected heroin	17 (100)	6 (100)	23 (100)
Injected heroin ≥ 4 times/week	11 (65)	6 (100)	17 (74)
Injected cocaine	2 (12)	1 (17)	3 (13)
Injected speedball (heroin and cocaine together)	2 (12)	0	2 (9)
Injected oxycodone	1 (6)	0	1 (4)
# of drugs used through non-injecting routes (excl. alcohol and marijuana)	1.5	2.3	1.7
Median years injecting (mean; range)	6 (11.9; 0.2–48)	4.5 (6.2; 2–16)	6 (10.4; 0.2–48)

We identified three major themes in our analyses on which non-helpers differed from helpers in their respective ideas about initiating others: altruistic motivations to prevent *both* immediate and delayed harms; negative assessments of one’s injection skills; and absolutist ethical reasoning against initiating others. Theory and prior research did not lead us to anticipate this third theme, which only became apparent during our analyses. We present quotations that best illuminate each theme, and how non-helpers differed from helpers. Bracketed information following each quotation indicates the participant’s gender, age (by decade), and number of years injecting. Participants sometimes conflated helping with first injections with helping *current* PWID with their injections. The interviewers would clarify this distinction with interview participants as needed. In all quotations that we use, whenever the term “helping” appears, the referenced behavior is helping with *first* injections rather than more general helping. Because we want to highlight the perspectives of the non-helpers, who also formed the majority of our sample, most quotations we cite come from them.

#### Altruistic motivations: preventing immediate and delayed harms

Non-helpers expressed altruistic motivations to prevent both immediate and more delayed harms to would-be injectors by not initiating them into injection drug use. We classify as immediate those harms that are directly evident while assisting with someone’s first injection, such overdosing and hitting an artery. We classify as delayed any harms that occur subsequent to the initial injection period, both acute and those unfolding more gradually, such as becoming addicted, HCV and HIV infection and related illnesses, habitual criminal behavior, deteriorating social networks, and worsening employment, housing, and educational opportunities.

##### Non-helpers

Participant: No. I would not inject another person. I would not want the chance of missing and giving them an abscess. And I am not going to be held responsible for nobody. [male, 20s, injecting 2 years]

Participant: I would not feel right about it. Especially if I give it to some person that shot and they OD’d in front of me. That’s not such a good idea. You know? Some people, you do not know how strong stuff is or what kind of, a person could have a heart problem or something wrong with them… [male, 60s, injecting 30 years]

Participant: I tell them [non-injectors] how negative it is and how they’ll get addicted to the needle itself… And that once they get addicted to that needle, how much more problems they are going to have and chances of dying, too. Like overdose and everything else. [male, 50s, injecting 34 years]

Participant: I refuse to [help inject others for the first time]… people have asked me to, you know, can you shoot me up? And I’ll say no. Because once you start, you are not going to stop. And your life will turn right around, and you’ll lose everything. It’s so true. You will lose everything. And I refuse to do that to somebody. [female, 20s, injecting 2 years]

Participant: I would not want somebody to be a shooter now because I showed them how to shoot. No. I do not do it. I keep telling people keep sniffing it ‘cause shooting has so many risks for health issues now that it’s just not even worth it. If you do not know what you are doing, or if you are not body conscientious, you are gonna wind up with HIV or something soon. It’s only a matter of time… [female, 30s, injecting 3 months]

Motivated by altruistic concerns, non-helpers describe numerous immediate and delayed harms to would-be injectors that they, as non-helpers, want to avert, either directly in the short term or indirectly in the long term. Two participants describe a fear of directly causing abscesses by missing a vein or of contributing to an overdose if they were to help a novice. Such concerns about causing immediate harms may stem from a lack of confidence in their skills injecting others, which we discuss below. By not helping, non-helpers may feel they can also play a role in preventing delayed harms. Thus, non-helpers suggest they may have prevented an addiction to the needle, a deepening drug dependency, becoming infected with HIV, and “losing everything.” Notably, helpers were as likely as non-helpers to describe altruistic motivations for their choice to help.

##### Helpers

Interviewer: What led you to helping them inject?

Participant: ‘Cause she could not do it. Plus it’s dangerous. If you do not know what you are doing, it’s dangerous. You could hit in a nerve, you could hit an artery. You could miss, your arm could swell up. [male, 30s, injecting 3 years]

Participant: The positive thing is that I can help them not – first of all not get Hep C, do it right, you know what I am saying, where they cannot hurt themselves, because nobody knows that – the different type of veins that you are not supposed to hit, you know, inject anything into it. You can tell the difference because if you are in a spot that you are not supposed to be in it’s a light red, but if you are in a spot where it’s the main line it’s like a burgundy… [male, 30s, injecting 2 years]

Non-helpers and helpers alike express altruistic motivations. Non-helpers view *not helping* as a strategy to prevent harm: preventing immediate harms because they often thought they lacked the skills to inject someone safely, and preventing delayed harms because they may have deterred the would-be injector from starting to inject. By contrast, helpers generally describe *helping* to prevent immediate harms based on their belief that they could inject the novice more safely than the novice him or herself could, or than another PWID could. References to preventing delayed harms are relatively absent in helpers’ accounts in our sample, presumably because the immediate harms they think they prevented are more salient to them and/or they do not think they played a role preventing delayed harms. As well, the non-helpers in our sample, who on average have injected for longer than the helpers, may have greater exposure to and recognition of the delayed harmful consequences of injecting, making those harms more salient to them. That altruism leads to opposite choices between non-helpers and helpers may also speak to the moral complexity of helping, since non-helpers and helpers alike cannot know with certainty the sequelae of their respective choices.

#### Skills

Implicit and sometimes explicit in many non-helpers’ accounts is a lack of confidence in their injection skills, including their skill injecting others. This lack of confidence in injecting skills among non-helpers in our sample occurs even among those with long histories of injecting, including in the two examples given below. When paired with altruistic motivations, non-helpers’ assessments that they lacked skills to safely initiate someone into injection led them to not help.

##### Non-helpers

Participant: I am not really that great at it. So I would not be the person to really do that… I am not great at that whole needle thing. I do it and I am lucky that I get mine. But I am not one of those injectors that like know, like I can be a phlebotomist. [female, 30s, injecting 14 years]

Participant: I wasn’t even good at myself, so I could not really help somebody… I did not feel like I would do it well for them…. I do not want to be the reason for anybody else’s mishaps. [male, 40s, injecting 13 years]

##### Helpers

Participant: A lot of them [people the participant has helped] are friends of mine from when I was younger. A couple of them are from when I met my drug dealer. They would be like, “Can you help me out? I really don’t know what I am doing.” A lot of them said that they do not know how to find their veins. For some reason, I can find anybody’s vein. I do not know why, but I feel like I am a doctor and I can catch somebody’s vein. That is really how I help people get there. [male, 20s, injecting 8 years]

Participant: I felt like – some of my friends I just felt like if they want to do it on their own I do not want them to mess up or like hurt themselves doing it and I knew how to do it, so I would just do it for them so they would not go out and have somebody else that did not know what they were doing… [female, 20s, injecting 6 years]

None of the non-helpers in our sample gives a positive assessment of their skills injecting others, whereas four of the six helpers do. Negative assessments of their own injection skills is integral to many non-helpers’ altruistic concerns about causing harm to a novice by helping with first injections. Because non-helpers had been injecting for a longer time period than helpers, these negative assessments are not from lack of experience. The assessments could be valid, or could reflect underestimates of their skills relative to others’ skills, overestimates of the risks, particularly strong aversions to taking those risks, or a composite across these dimensions. Thus, among two PWID in the same injection scenario and with the same injection skills, the more risk averse PWID may assess their skills relatively negatively. In short, self-assessments of one’s injection skills may be influenced by factors in addition to one’s actual injection skills.

#### Absolutist ethical reasoning

Non-helpers are frequently emphatic, and strikingly emotional, about not helping, regardless of the circumstances and potential for immediate harm to the novice injector. This suggests an absolutist form of ethical reasoning: making ethical choices based on a priori principles applied globally, regardless of situational factors. This contrasts with a more situational approach to ethics, where the particular facts of a situation influence or determine the proper course of action. The distinction between absolutist and situational forms of ethical reasoning has a long and rich history in ethical theory [56]. In the West, the former has roots in ancient Judaism and Christianity [57] and is expressed in Immanuel Kant’s “categorical imperative” [56]. Situational approaches to ethics are rooted in utilitarianism but are more recently anchored in the twentieth century work of Joseph Fletcher, who explicitly developed a theory of situational ethics [58]. Previous studies on initiating others into injection did not lead us to anticipate this theme, which only became apparent during our analysis of the text.

##### Non-helpers

Participant: It was done to me and that was like, that’s the Devil to me. The person that put it in my arm I look at as the Devil… Multiple people have asked me to inject them, including my girlfriend. I don’t care if they can’t find the vein or what they have to do it in. I just won’t put a needle in anybody’s arm or any vein or muscle. [male, 50s, injecting 34 years]

Participant: I would not do it… ‘Cause I despise the person that did it for me, and I do not have a hurtful bone. … There’s no real why, why not... Knowing what it can do to them, I would not do that. I do not want to be responsible for helping anybody with that. No way. [male, 40s, injecting 1 year]

Participant: I am a strong believer in karma. And I just refuse to do that to anybody, turn them on to something like that. Me and my boyfriend always say that. Never, I never have, and I never will… I am like, nope, no amount of money, no amount of heroin could ever make me do that and live with the guilt of doing that to somebody. Sorry. [female, 20s, injecting 2 years]

Participant: … sometimes the only thing I am going to say is I am not going to help you. To find somebody else, which they basically do anyway. But, I am not going to be the one to help them destroy their lives, bad… I cannot pinpoint why I do not feel right. [male, 60s, injecting 30 years]

Participant: I could not live with myself showing somebody how to do that. I would not want somebody to be a shooter now because I showed them how to shoot. No. I do not do it. [female, 30s, injecting 3 months]

Interviewer: And if a friend was doing a bad job [shooting themselves up the first time], would you care?

Participant: Absolutely, yeah… I would not shoot them up though. I would feel bad. [male, 20s, injecting 1 year]

##### Helpers

In contrast, helpers’ discussion of initiating others typically follows a situational rationale: because novice injectors lack injecting skills; because their transition to injection is inevitable; or because they will injure themselves if an experienced injector does not help, therefore I, an experienced injector, will help them. Had any of these factors not been present, helpers may have chosen not to help.

Participant: … if they are going to do it and they do not know what they are doing, then I will help you, because I do not want you to die and I do not want you to mess up yourself. [male, 20s, injecting 8 years]

Interviewer: How do you feel now in hindsight that you helped them inject the first time?

Participant: Nah, they would’ve eventually did it on their own. They would’ve eventually did it basically. [male, 30s, injecting 3 years]

For some non-helpers, even if assisting with a first injection averts immediate harm, and even if the person’s transition to injection were inevitable, superordinate long-term harms loomed and they indicated a moral aversion to playing a causal role in those harms. Such aversion was often expressed in strongly emotional terms. This emotion was sometimes driven by contempt for the person who had initiated them, who two helpers above described as “the devil” and someone they “despised.” For these two, their own regrets about initiating injection drug use seem indelibly linked to the person who initiated them. This is meaningful because, to the degree PWID morally implicate those who helped with their *own* initiation into injection, they may be deterred from helping with *others’* first injections to avoid self-reproach. And, many non-helpers expressed not wanting to feel guilty or responsible for another person’s decline. One could view this approach as a defense of moral respectability, as guilt avoidance, or simply as altruism. Whatever the precise motivation, non-helpers often spoke in injunctive terms against helping. With a more immediate temporal focus, helpers attended to preventing imminent harm. Implicit in many helpers’ accounts is that they chose to help *when* injection seemed inevitable *and* they thought they could minimize immediate harm.

## Discussion

Over the past 10 years of the USA opioid epidemic, marked shifts have occurred—from pharmaceutical opioids to heroin and from non-injecting to injecting. To examine these phenomena in a hotspot of the epidemic, we conducted qualitative interviews with 23 PWID on Staten Island in New York City. Most novice PWID need experienced PWID to help them with their first injection, making this dyad crucial to the sustenance, and expansion, of injection across drug-using cohorts. Therefore, one goal of our interviews was to learn about PWIDs’ current ideas about initiating others into injection. Research over the last 20 years has begun to uncover the dynamics of initiation, from the perspectives of both the initiate and the experienced PWID who assists. Perspectives on initiating from the majority of PWID who have never done so are under-represented in the literature. An enhanced understanding of the cognitive, behavioral, and emotional underpinnings of the norm not to help may be relevant to harm reduction interventions to reduce PWIDs’ promotion of injection drug use.

Three main themes emerged from the discourse on initiating others into injection drug use among the 17 non-helper PWID in our sample. First, non-helpers expressed altruistic motivations to prevent both immediate and delayed harms to new injectors. Second, non-helpers gave negative assessments of their skills injecting others, thus making assistance an opportunity to cause rather than prevent immediate harms to the initiate. Third, non-helpers employed what we interpret as an ethically absolutist view against helping in which situational factors are irrelevant to their choice. We interpret these three themes as mutually reinforcing: together they create a stronger rationale for not helping than any one theme alone. The non-helpers differed from the six helpers in our sample on all three themes. Helpers focused on preventing immediate harms by *helping* and rarely addressed preventing delayed harms. They assessed their skills injecting others more favorably than did non-helpers. Lastly, helpers’ rationales for helping followed a situational *if-then* course.

To the best of our knowledge, of the three themes we identified among non-helpers in our sample, only altruism has been mentioned previously in the few reports of non-helpers’ perspectives on helping. Wenger et al. [35] describe non-helpers not wanting to introduce people to injection, given the harmful immediate and delayed consequences. Some non-helpers would alternatively teach non-injecting drug users safer injection practices while stopping short of helping them inject. Other research [33, 37] described social codes against initiating *young* persons into injection drug use, while also indicating how frequently the code was ignored. In our sample, non-helpers did not mention teaching safer injection to would-be injectors nor did they describe a *social* code against helping (though they frequently expressed *personal* codes against it). When we asked about future intentions to assist with first injections, regardless of whether their intentions were to help or not to help, participants seemed more influenced by their personal attitudes (e.g., their altruistic desire to prevent harm) than by their perceptions of social norms. Thus, in view of the theory of reasoned action’s proposition that behavioral intentions are influenced both by one’s own attitudes and perceived norms among others, we note that attitudes predominate in our sample. The relative absence of social norms in our participants’ discourse on helping could signal that such norms have not had time to develop in the possibly still maturing drug-using cohort from which we drew our sample.

Previous evidence [33–35] of PWIDs’ ambivalence about helping with others’ first injections has largely been derived from samples who have initiated others into injection in the past. Informed by the decisional balance model [50], we pursued the role of ambivalence, and the tension between the perceived pros and cons of helping, in understanding motivations to assist with others’ first injections. This was useful in distinguishing between the experiences of helpers and non-helpers. We encountered minimal ambivalence about helping among the non-helpers in our sample, for whom the cons of helping far outweigh the pros. Their lack of ambivalence is evident throughout the quotations we cite in our results. By comparison, the helpers in our sample express ambivalence about helping, which is consistent with prior findings [34, 35]. However, in terms of the decisional balance model [49, 50], helpers primarily describe the pros of helping whereas non-helpers focus on the cons. Our data suggest that participants had clearly thought about the issue of helping others and that they had arrived at a decisional balance that reflects their behaviors.

The theme of injection initiation occurring within sexual partnerships arose minimally in our sample, compared with other samples [30, 55]. The six helpers mentioned helping friends, acquaintances, and strangers; only one helper mentioned helping someone who could plausibly be identified as a sex partner. One non-helper described *not* helping a sexual partner with her first injection, saying “I don’t care if they [i.e., his girlfriend and other people he has refused to help] can’t find the vein or what they have to do it in. I just will not put a needle in anybody’s arm or any vein or muscle.”

We attribute the lack of parallel findings across studies—not only regarding sexual partnerships providing the context of injection initiation, but also the two new themes we identified among the non-helpers in our sample—to different study foci, small sample sizes, and potential differences over geographic contexts and time.

### Implications for interventions—how lessons from non-helpers might be applied to current helpers

Interventions with PWID who are currently helping with others’ first injections, or are inclined to, can be firmly grounded in harm reduction principles. Though these interventions can be aimed at motivating PWID to refrain from assisting with first injections, these interventions can simultaneously be designed to affirm the autonomy of PWID to arrive at conclusions consistent with their own principles around helping. A preliminary goal of such interventions, then, is to give PWID the opportunity to discuss and clarify these principles—an opportunity they may not have in their everyday lives. This discussion could frame the choice between helping versus not helping as also a choice about *how* to help or not help. Findings from our study suggest numerous approaches, which we discuss immediately below, to not only motivate PWID to refrain from assisting with first injections, but also to support future helpers and non-helpers in reducing harms to those who are curious about injecting (e.g., how to avoid abscesses and disease transmission).

PWID who are motivated to help by focusing on preventing immediate harms can be asked to consider how injection exposes persons to the risk of profound harms in the long run. Further, to the extent PWID think they can play a causal role in preventing immediate harms, they might consider the additional possibility of playing a role in preventing delayed harms by not assisting. If they believe transitions to injection are inevitable, they could consider whether presumed inevitability necessarily renders their assistance ethically neutral. PWID also could be asked to realistically assess their skills injecting others, in light of the risks for complications and their level of risk aversion. The opportunity to weigh their skills more deliberately and in a larger framework may lead to more nuanced estimations. PWID could be encouraged to consider how they apportion responsibility for their own initiation into injection. To the extent they regret injecting, and implicate a PWID who may have helped with their own first injection, they could be asked to consider whether they would be likely to seriously regret having helped with someone else’s first injection. As suggested in other samples [35], PWID could view requests for help with first injections not merely as opportunities to help or not to help, but also as opportunities to have an honest discussion about what injection is like, share personal experiences of harms from injecting, impart safer injection practices, make referrals and introductions to community-based resources, and to provide immediate social support. In sum, interventions to motivate PWID to refrain from initiating others into injection can invite a broad discussion not only just about helping versus not helping, but also about helping and not-helping to reduce harm to the non-PWID.

Ultimately, we recognize that because PWID who contemplate initiating someone into injection cannot know with certainty the sequelae of either choice (helping and not helping), the question of helping with a first injection can be ethically complicated. There is the possibility that not helping or helping may lead to more or less harm to the person who may inject for the first time. There is also the possibility that helping with a first injection may lead to more or less harm to the person who helps—the regret expressed by helpers for having helped can be considered as evidence of harm to the helper, particularly if helping violated the moral code of the helper. Given the uncertainties in the outcomes for helping or not helping in specific instances, it may not be possible to determine whether helping or not helping is the most ethical decision in all of these instances. It is also likely that there will be some instances where helping with a safer first injection may be considered the ethical choice. Our participants present two alternatives for making ethical choices in situations where outcomes cannot be known with certainty. Helpers tended to make assumptions about harm coming to at least some would-be injectors if they did not help, and therefore they helped. Non-helpers, on the other hand, tended to make the assumption that injecting inevitably led to harm and that therefore it was inherently wrong to help. Thus, a potential lesson for PWID, and for relevant interventions, is that PWID can decide a priori which of two PWID “groups” they fit into, which roughly correspond to situational and absolutist approaches: those who assess immediate risks and attempt to prevent those that seem possible or likely, and those who think injecting is inherently wrong because it inevitably causes great harm, and therefore they will not do it. In any case, there is a critical need for future research to address the information gap on what happens when non-PWIDs’ requests for help with first injections are refused. To what extent do non-PWID in this situation refrain both from self-injecting and from seeking others’ help? Data addressing these questions could shed light on the extent to which refusals to help with first injections lead to risky injection, or delay or forestall injection initiation.

#### Strengths and limitations

Whereas previous studies have focused on the predictors and psychosocial contexts of helping with first injections, our study is the first we are aware of that highlights the voice of the majority of PWID who have never helped with a first injection. Moreover, our sample is drawn from a hotspot of the continuing opioid epidemic in the US. Nevertheless, our sample is small, local, and non-representative of PWID in general. Our mode of data collection was a single interview – eliciting retrospective and current accounts of highly charged topics, especially injection and initiating others into injection. There is potential for minimization, distorted recall, and social desirability bias in which helping behaviors are under- or not reported, leading to some possible misclassification in our sample. As well, some non-helpers in our sample could help in the future, and some did not rule out the possibility, and some helpers expressed commitments to not helping in the future. However, the proportion in our sample who said they had helped others with a first injection is consistent with other studies, suggesting that helping is the province of a minority of experienced injectors. Moreover, findings that helpers often assisted with more than one first injection [25, 28–32] are consistent with the idea that some experienced injectors play a disproportionate role initiating others into injection, effectively “compensating” for those who never help with first injections. In short, we are confident that most in our sample, if not all, who state they have never helped with a first injection are reporting accurately, and that misclassification on this factor is minimal.

## Conclusions

Among the 17 PWID in our sample who have never helped someone with their first injection, three themes arose in their discourse on not initiating others into injection: not helping because of altruistic motivations to prevent immediate *and* delayed harms; not helping because they lacked skills injecting others; and an absolutist prohibition on initiating others into injection. These themes were not echoed among the helpers in our sample. In nearly all PWID samples, non-helpers comprise a majority, suggesting that their perspectives on initiating others have considerable implications for public health. Peer-driven harm reduction interventions to reduce the role PWID play fostering injection, or to encourage PWID to promote others’ safer injection initiation, can integrate these themes into discussions with PWID about their future intentions around promoting injection and helping with first injections.
